# YAP and TAZ in Vascular Smooth Muscle Confer Protection Against Hypertensive Vasculopathy

**DOI:** 10.1161/ATVBAHA.121.317365

**Published:** 2022-02-24

**Authors:** Fatima Daoud, Marycarmen Arévalo Martinez, Johan Holmberg, Azra Alajbegovic, Neserin Ali, Catarina Rippe, Karl Swärd, Sebastian Albinsson

**Affiliations:** Department of Experimental Medical Science (F.D., M.A.M., J.H., A.A., C.R., K.S., S.A.), Lund University, Sweden.; Department of Clinical Sciences Lund, Orthopaedics, Clinical Epidemiology Unit (N.A.), Lund University, Sweden.

**Keywords:** adventitia, compliance, hyperplasia, muscle, smooth, vascular, neointima

## Abstract

Hypertension remains a major risk factor for cardiovascular diseases, but the underlying mechanisms are not well understood. We hypothesize that appropriate mechanotransduction and contractile function in vascular smooth muscle cells are crucial to maintain vascular wall integrity. The Hippo pathway effectors YAP (yes-associated protein 1) and TAZ (WW domain containing transcription regulator 1) have been identified as mechanosensitive transcriptional coactivators. However, their role in vascular smooth muscle cell mechanotransduction has not been investigated in vivo.

**Methods:**

We performed physiological and molecular analyses utilizing an inducible smooth muscle–specific YAP/TAZ knockout mouse model.

**Results:**

Arteries lacking YAP/TAZ have reduced agonist-mediated contraction, decreased myogenic response, and attenuated stretch-induced transcriptional regulation of smooth muscle markers. Moreover, in established hypertension, YAP/TAZ knockout results in severe vascular lesions in small mesenteric arteries characterized by neointimal hyperplasia, elastin degradation, and adventitial thickening.

**Conclusions:**

This study demonstrates a protective role of YAP/TAZ against hypertensive vasculopathy.

**Highlights**

- YAP (yes-associated protein 1) and TAZ (WW domain containing transcription regulator 1) regulate genes involved in vascular smooth muscle differentiation and contraction.
- Smooth muscle–specific deletion of YAP/TAZ leads to reduced vascular contractility, impaired myogenic response, and increased compliance.
- YAP and TAZ are crucial for adaption to elevated mechanical stress and protection against hypertension-induced vascular lesions.

Vascular smooth muscle cells actively interact with the surrounding microenvironment, and they are continuously exposed to hormonal factors and hemodynamic forces. Vascular smooth muscle cells respond to such hemodynamic forces and regulate blood pressure and flow. In small resistance arteries, an acute increase of intraluminal pressure induces a myogenic response.^1^ However, prolonged periods of sustained elevation of blood pressure may trigger pathological inward remodeling.^2^ Therefore, an appropriate response to mechanical stimuli in the vascular wall is essential to reduce the risk of hypertension-induced vascular diseases. Hypertension is a major risk factor for cardiovascular diseases, which are the leading cause of mortality in the developed countries.^3,4^ Importantly, studies have shown that ≈50% of patients with hypertension have uncontrolled hypertension.^5^ This is partly due to resistance to treatment, which is defined as persistent high blood pressure despite administration of ≥3 antihypertensive medications.

Despite the well-established association between hypertension and vascular diseases, the mechanistic link between increased blood pressure and pathological changes in the vascular wall is not well understood. Identifying factors that influence mechanical damage is, therefore, crucial to understand hypertension-induced vascular diseases and could help identifying novel therapeutic targets to treat these life-threatening conditions. We hypothesize that appropriate mechanotransduction and contractile function in vascular smooth muscle are crucial factors for maintaining vascular integrity during elevated systemic blood pressure levels. A perturbed ability to sense or adapt to elevated mechanical stress may result in vascular injury and trigger repair mechanisms that involves neointimal hyperplasia and adventitial fibrosis.

YAP (yes-associated protein 1) and TAZ (WW domain containing transcription regulator 1) are effectors of the Hippo signaling pathway that control organ size during development. YAP and TAZ have also been recognized as mechanosensitive transcriptional coactivators.^6^ They respond to substrate stiffness, cell density, and shear stress by shuttling between the cytoplasm and nucleus. In the nucleus, YAP/TAZ mainly binds to TEAD (TEA domain transcription factor) and influences gene expression regulating cell proliferation, differentiation, and apoptosis.^7^ When the inhibitory kinases of the Hippo pathway are active, YAP/TAZ is phosphorylated and sequestered in the cytoplasm. Independently of the Hippo pathway, YAP/TAZ can be activated by Rho GTPases and actin polymerization.^8,9^ YAP has also been shown to interact with the actin-sensitive MRTFs (myocardin-related transcription factors), particularly MRTF-B.^10,11^

Constitutive YAP deletion in mouse cardiomyocytes and vascular smooth muscle cells using transgelin (SM22α) Cre recombinase results in perinatal lethality.^12^ These mice exhibit various degrees of cardiac abnormalities, as well as aberrant vascular structure characterized by wall thinning due to decreased smooth muscle proliferation. We recently demonstrated that inducible deletion of YAP/TAZ in adult smooth muscle causes rapid lethality within 2 weeks due to colonic pseudo-obstruction,^13^ emphasizing the importance of YAP/TAZ in maintaining the contractile phenotype of smooth muscle. Y/T KO (YAP/TAZ knockout) mice displayed a downregulation of several smooth muscle markers, including *Myh11*, *Acta2*, *Cnn1*, and *Tagln*, along with reduced contractility and peristalsis in the colon. We also found a reduction of the muscarinic receptors M_2_ and M_3_ (*Chrm2* and *Chrm3*), and this likely contributes critically to the reduced contractility. A very recent study reported similar findings of reduced smooth muscle marker expression and contractility in the vasculature upon YAP/TAZ loss.^14^

In this study, we investigate the functional role of YAP/TAZ in vascular smooth muscle. We show that deletion of YAP/TAZ in vivo results in impaired agonist-stimulated contractility and myogenic responsiveness, reduction of mechanically induced gene expression, and increased distensibility. These defects associate with the development of severe vascular lesions in Y/T KO small mesenteric arteries upon angiotensin II–induced hypertension.

## Materials and Methods

The RNA sequencing (RNA-seq) data have been made publicly available at the Gene Expression Omnibus repository and can be accessed at https://www.ncbi.nlm.nih.gov/geo/query/acc.cgi?acc=GSE180631. Additional data that support the findings of this study are available from the corresponding author upon reasonable request.

### Animal Procedures

The *Wwtr1^tm1Hmc^* *Yap1^tm1Hmc^*/WranJ and the *Myh11*-Cre/ERT2 mice were described previously (The Jackson Laboratory; #030532 and #019079).^13,15,16^ Following anesthesia (2% isoflurane), osmotic minipumps (Alzet, model 2004) loaded with angiotensin II in saline solution (1 mg/kg per day) were implanted subcutaneously in mice at 4 to 6 weeks of age.^17^ Angiotensin II treatment started 14 days before the first tamoxifen administration. Conditional gene knockout of YAP and TAZ was achieved by intraperitoneal injections of tamoxifen for 5 consecutive days (1 mg/mouse per day).^13^ The Cre recombinase transgene is positioned on the Y chromosome, and consequently, only male mice were used in this study. Tamoxifen-treated *Myh11*-Cre/ERT2–negative *Yap1*/*Wwtr1* floxed mice were used as control unless otherwise indicated. Systolic and diastolic blood pressures were measured in conscious mice using a tail cuff system (CODA System; Kent Scientific). To minimize stress and decrease variability, the mice were trained 3× every second day for 1 week before the measurements. Blood pressure was recorded both before pump implantation and at the end of the experiment. Fifteen cycle measurements were collected, and the mean arterial pressure was calculated for evaluation of arterial blood pressure.

B6.129(Cg)-*Gt*(*ROSA)26Sor^tm4(ACTB-tdTomato,-EGFP)Luo^*/J mice were used as Cre reporter strain as described previously (The Jackson Laboratory; #007676).^13,18^ Briefly, the ROSA^mT/mG^ mice were bred with *Myh11*-Cre/ERT2 to establish the ROSA^mT/mG^-Cre strain, which was then crossed with YAP^fl/fl^/TAZ^fl/fl^ females. YAP^fl/fl^/TAZ^fl/fl^ Cre-negative ROSA^mT/mG^ were used as controls. The reporter mice were only used for lineage-tracing experiments. All animal procedures were performed in accordance with the Malmö/Lund Committee for Animal Experiment Ethics, Lund, Sweden (M6-15, M61-16, and M2990-20).

### Histological Analysis

Aortae and mesenteric arteries were dissected and incubated in 4% formaldehyde overnight and 1 hour, respectively, at 4 °C followed by immersion in 20% sucrose for 24 hours at 4 °C and frozen in optimal cutting temperature (Tissue Tek-Sakura Finetek; #4583).

### Elastin Staining

Elastic connective tissue stain kit (Abcam; #ab150667) was used according to the manufacturer’s recommendations. Cryosections (8 µm) were incubated with the elastic stain solution for 15 minutes, washed with tap water, and immersed in differentiating solution for 1 minute and 20 seconds. Next, the slides were washed in running tap water and incubated in sodium thiosulfate solution for 1 minute followed by incubation in Van Gieson solution for 2 minutes, dehydrated with absolute ethanol, and cleared with xylene. Finally, slides were mounted with Pertex mounting medium (HistoLab; #5500552).

### Oil Red O Staining

Oil Red O was used to detect fat. Slides with tissue sections were fixated in 4% formaldehyde for 60 minutes, washed in water, and incubated in Oil Red O working solution (0.5% oil red [Sigma; #O0525] dissolved in isopropanol, filtered with #3 filter paper, diluted in MilliQ H_2_O, and filtered in 0.2 µm syringe filter). Slides were washed with distilled water and running tap water for 3 minutes. Harris hematoxylin (HistoLab; #01800) was used to stain nuclei. Finally, sections were washed with distilled water and mounted with 50% glycerol in H_2_O.

### Alizarin Red Staining

Cryosections were incubated in 2% Alizarin Red (Sigma-Aldrich; #A5533-25G) in distilled H_2_O with pH 4.2 for 2 minutes followed by dehydration in 100% acetone and 1:1 solution of acetone:xylene. Finally, sections were cleared with xylene and mounted with Pertex (HistoLab; #5500552).

### Imaging

Pictures were obtained with Aperio ScanScope Console, version 10.0.0.0 (Aperio Technologies, Inc, Vista, CA), with ×20 magnification.

### Immunofluorescence

Cryosections (8 µm) were permeabilized with 0.2% TritonX-100 in PBS for 10 minutes and incubated with blocking solution (3% BSA in PBS) for 30 minutes. Samples were incubated with primary antibodies against Ki67 (Abcam; #ab15580, 1:100), YAP/TAZ (Cell Signaling; #8418, 1:200), and MAC2 (galectin-3; Cedarlane; #CL8942AP, 1:200) overnight at 4 °C followed by secondary antibody incubation for 1 hour at room temperature (Invitrogen; #A21428, #A11081, or #A21422, 1:500). Finally, nuclei were labeled with DAPI (4′,6-diamidino-2-phenylindole; 1 µg/mL), and slides were mounted with Aqua-Poly/Mount (Polysciences Europe; #18606-20). Pictures were obtained with an Olympus BX60 microscope in combination with an Olympus DP72 camera using the CellSens Dimension software.

### Wire Myography

Wire myography experiments were performed using 4-channel myograph systems (models 620M and 610M; Danish Myo Technology). Two different protocols were used. In the first protocol, we used tail arteries from 4 groups of mice; Cre-negative, Y/T KO, Cre-negative plus angiotensin II, and Y/T KO plus angiotensin II. The vessels were set to 5 mN preload tension and equilibrated for 40 minutes in Ca^2+^ containing HEPES-buffered Krebs solution at 37 °C (135.5 mmol/L NaCl, 5.9 mmol/L KCl, 1.2 mmol/L MgCl_2_, 11.6 mmol/L HEPES, 11.5 mmol/L glucose, and 143.8 mmol/L Cl^−^). Vessels were then stimulated with 60 mmol/L KCl twice. Dose-response curves for AVP (arginine vasopressin; Tocris; #2935), 5-HT (5-hydroxytryptamine/serotonin; Sigma-Aldrich; #H4511-500MG), cirazoline hydrochloride (Tocris; #0888), and U 46619 thromboxane A_2_ receptor agonist (Tocris; #1932) were generated. Force measurements were normalized to the length of the vessels and expressed as mN/mm. In the second protocol, abdominal aortae of vehicle and tamoxifen-treated *Myh11*-CreER^T2^ *Yap1*^fl/fl^ *Wwtr1*^fl/fl^ mice were mounted, and increments in circumference were done in HEPES-buffered Krebs solution at 37 °C. This was followed by a period of equilibration in nominally Ca^2+^-free solution where the passive force was recorded at the end.

### Pressure Myography

Experiments were performed as described previously.^19^ Briefly, 2 segments of second-order mesenteric arteries from each mouse were dissected and cannulated on thin glass pipettes in an organ bath. The temperature was maintained at 37 °C throughout the experiment. The vessels were stretched longitudinally and allowed to equilibrate at 45 mm Hg for 40 minutes in HEPES-Krebs buffer with Ca^2+^. Outer diameter was continuously monitored using a video camera and edge detection software. After 60 mmol/L KCl stimulation, the intraluminal pressure was increased stepwise from 20 to 120 mm Hg to evaluate myogenic tone. The intraluminal pressure was then set to 45 mm Hg, and a transient contraction was induced using a single dose of angiotensin II (100 nmol/L; Tocris; #1158). Analysis of myogenic tone was then performed in the presence of angiotensin II. At the end of each experiment, the buffer was replaced by Ca^2+^-free buffer supplemented with 2 mmol/L EGTA to measure the passive diameter at the corresponding pressure. Active and passive vessel diameters were measured in the presence and absence of Ca^2+^, respectively. The myogenic contraction was calculated by subtracting the active diameter from the passive and dividing it by the passive diameter.

### Portal Vein Model

Portal veins were dissected free from the surrounding tissue, connected to a hook at one end and the other end kept free or stretched with 0.3 g gold weight as described previously.^20^ Portal veins were incubated in DMEM Ham’s F12 (VWR; #L0092-500), 2% dialyzed fetal bovine serum (Thermo Fisher Scientific; #A3382001), 50 U/50 µg/mL penicillin/streptomycin (Biochrom; #A2212), and 10 nmol/L insulin (Sigma-Aldrich; #I-5500) at 37 °C, 5% CO_2_. After 24 hours, portal veins were snap-frozen in liquid nitrogen.

### Protein Extraction and Western Blotting

Abdominal aortae of Cre-negative (control) and Y/T KO mice were homogenized in SDS sample buffer. Protein determination was done using Bio-Rad DC protein assay. Equal amount of protein was loaded onto 4% to 15% Criterion TGX (Bio-Rad) gels. Following electrophoresis, proteins were transferred either by semidry Trans-Blot Turbo System (Bio-Rad) or by overnight wet transfer. Total protein was measured after transfer by Revert 700 Total Protein Stain kit (LI-COR; #926-11010) and used for normalization. Membranes were incubated overnight at 4 °C with primary antibody; YAP/TAZ (Cell Signaling; #8418, 1:1000), smooth muscle myosin heavy chain (Abcam; #ab53219, 1:1000), α-actin (Sigma-Aldrich; #A5228, 1:1000), PPARγ (peroxisome proliferator activated receptor gamma; Cell Signaling; #2443, 1:1000), ROCK1 (Rho kinase 1; Cell Signaling; #4035, 1:1000), C/EBPα (CCAAT enhancer binding protein alpha; Cell Signaling; #2295, 1:1000), or C/EBPβ (CCAAT enhancer binding protein beta; Cell Signaling; #3087, 1:1000). Either fluorescent or horseradish peroxidase-linked secondary antibodies were used. SuperSignal West Femto Maximum Sensitivity Substrate (Thermo Fisher Scientific; #34095) was used for chemiluminescent Western blot detection. Immunoblots were acquired using an Odyssey Fc instrument and analyzed using the Image Studio software (LI-COR Biosciences).

For PPARγ nuclear localization, Nuclear Extraction Kit (Abcam; #ab221978) was used to isolate the nuclear fraction of control or Y/T KO fresh aortae, according to the manufacturer’s instructions. The concentration of nuclear proteins was determined using the Bio-Rad DC protein assay followed by electrophoresis and overnight wet transfer. Protein level of histone H3 (Cell Signaling; #4499S, 1:1000) was used for normalization.

### Real-Time Reverse Transcription-Quantitative Polymerase Chain Reaction

Vessels were disrupted and homogenized using TissueLyser LT (Qiagen; #85600) followed by addition of QIAzol (Qiagen; #79306). Total RNA was isolated using the miRNeasy Mini Kit and QIAcube (both from Qiagen) according to the manufacturer’s protocol. One-step reverse transcription-quantitative polymerase chain reaction was performed using the StepOnePlus Real-Time PCR System (Applied Biosystems; #4376600). Individual mRNA targets were analyzed using commercially available primers from QuantiTect Primer assays (Qiagen).

### RNA-Seq and Analysis

Aortae were homogenized, and RNA extraction was done using RNeasy Mini kit (Qiagen; #74104) and QIAcube (Qiagen). RNA quality was assessed using the 2100 Bioanalyzer instrument (Agilent). Samples with RNA integrity number above 7 were chosen for further analysis. Library preparation was performed using the TruSeq kit (Illumina) and RNA-seq by NextSeq 500 platform (Illumina). RNA-seq data were analyzed using the DESeq2 package.

Gene ontology (GO) enrichment analysis was performed using the Protein Analysis Through Evolutionary Relationships (PANTHER) classification system (version 16; release date, December 18, 2020). The list of significantly (*P*<0.05) downregulated genes was uploaded, and the whole genome of *Mus musculus* was used as a reference list. Overrepresentation test with Fisher exact test with false discovery rate correction was used for statistical analysis. The annotation data sets were GO-Slim cellular component.

RNA-seq data were subjected to analysis using the Ingenuity Pathway Analysis software (Ingenuity Systems; www.ingenuity.com; November 30, 2020; Qiagen). A cutoff *P* of <0.05 was selected. The significant differentially expressed genes (724 upregulated and 540 downregulated) were compared with the whole data set consisting of 17 146 genes. The software was unable to map 603 genes, hence they were excluded from the analysis. Master regulator analysis was performed to predict the transcriptional regulators that may be the cause of observed changes in gene expression. For this prediction, a *Z* score was provided, and a positive *Z* score indicated activation, whereas a negative *Z* score indicated inhibition. The list was filtered to only include transcriptional regulators with matched *Z* score and fold change direction.

### Correlation Analysis

RNA-seq data were downloaded from the GTExPortal.org in June 2020 using R scripts as described.^21^ Correlations of transcript levels (in transcripts per million) were examined using the Spearman method in GraphPad Prism (San Diego, CA). Coronary artery, tibial artery, and aorta were examined. The number of individuals per tissue were 240 (coronary artery), 432 (aorta), and 663 (tibial artery).

### Statistical Analysis

All results are presented as mean±SEM. n values reflect the number of mice in each group. Continuous variables were assessed for normality by Shapiro-Wilk and homogeneity of variance by either F tests or Bartlett test. If data passed both tests, 2-sided unpaired Student *t* test was used for 2-group comparisons and 1-way ANOVA (1-way ANOVA) followed by Tukey for multigroup comparisons. If either normality or equal variance test failed, then Mann-Whitney *U* test was used for 2-group comparisons. Myography data were analyzed by repeated measures 2-way ANOVA followed by Bonferroni for multiple comparisons. Normality and equal variance were not assessed as a precondition for this analysis. *P*<0.05 was considered statistically significant. *P* values up to 3 decimal digits are depicted in figures. Statistical analyses mentioned above were performed using GraphPad Prism, version 9.1.0.

## Results

### Arteries Devoid of YAP/TAZ Demonstrate Reduced Contractile Responses to Various Vasoactive Agonists and Increased Vascular Compliance

**Figure 1.**
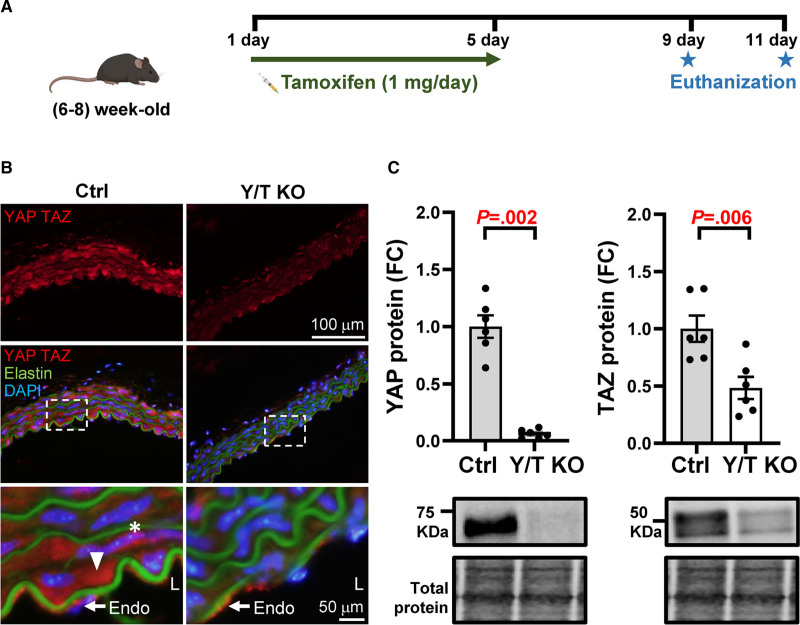
**Reduced expression of YAP (yes-associated protein 1) and TAZ (WW domain containing transcription regulator 1) in vascular smooth muscle. A**, Generation of Y/T KO (YAP and TAZ knockout) mice. *Myh11*-CreER^T2^ *Yap1*^fl/fl^ *Wwtr1*^fl/fl^ mice were injected with 1 mg tamoxifen for 5 consecutive days and euthanized on either day 9 or 11. **B**, Representative images of aortic cryosections, immunostained for YAP and TAZ. Asterisk represents nuclear localization, and arrowhead represents cytosolic localization. **C**, Western blot analysis, YAP and TAZ were normalized to total protein in abdominal aortic lysates (Ctrl [control], n=6; Y/T KO, n=6). The representative blots of YAP and TAZ are taken from the same lanes, hence the loading controls are identical. All data are presented as mean±SEM. DAPI indicates 4′,6-diamidino-2-phenylindole; Endo, endothelium; FC, fold change; and L, lumen.

*Myh11*-CreER^T2^ *Yap1*^*fl/fl*^ *Wwtr1*^*fl/fl*^ mice (henceforth referred to as Y/T KO mice) were injected with tamoxifen for 5 consecutive days (Figure 1A). Tamoxifen-treated Cre-negative mice were used as controls. As described previously, Y/T KO mice develop symptoms of intestinal pseudo-obstruction after ≈12 to 14 days.^13^ Therefore, we euthanized the mice at 9 or 11 days after the first tamoxifen injection. Immunohistochemistry of the aorta showed positive nuclear and cytoplasmic staining of YAP/TAZ in the smooth muscle layer of control mice, which was dramatically reduced in Y/T KO mice (Figure 1B). YAP/TAZ expression was clearly present in the endothelium of both control and Y/T KO aorta, as expected. Reduced expression of YAP/TAZ was also confirmed by Western blot analysis of abdominal aortic lysates (Figure 1C). The residual expression of TAZ in Y/T KO mice likely reflects expression in non–smooth muscle cells present in the aorta, such as endothelial cells and fibroblasts.

**Figure 2.**
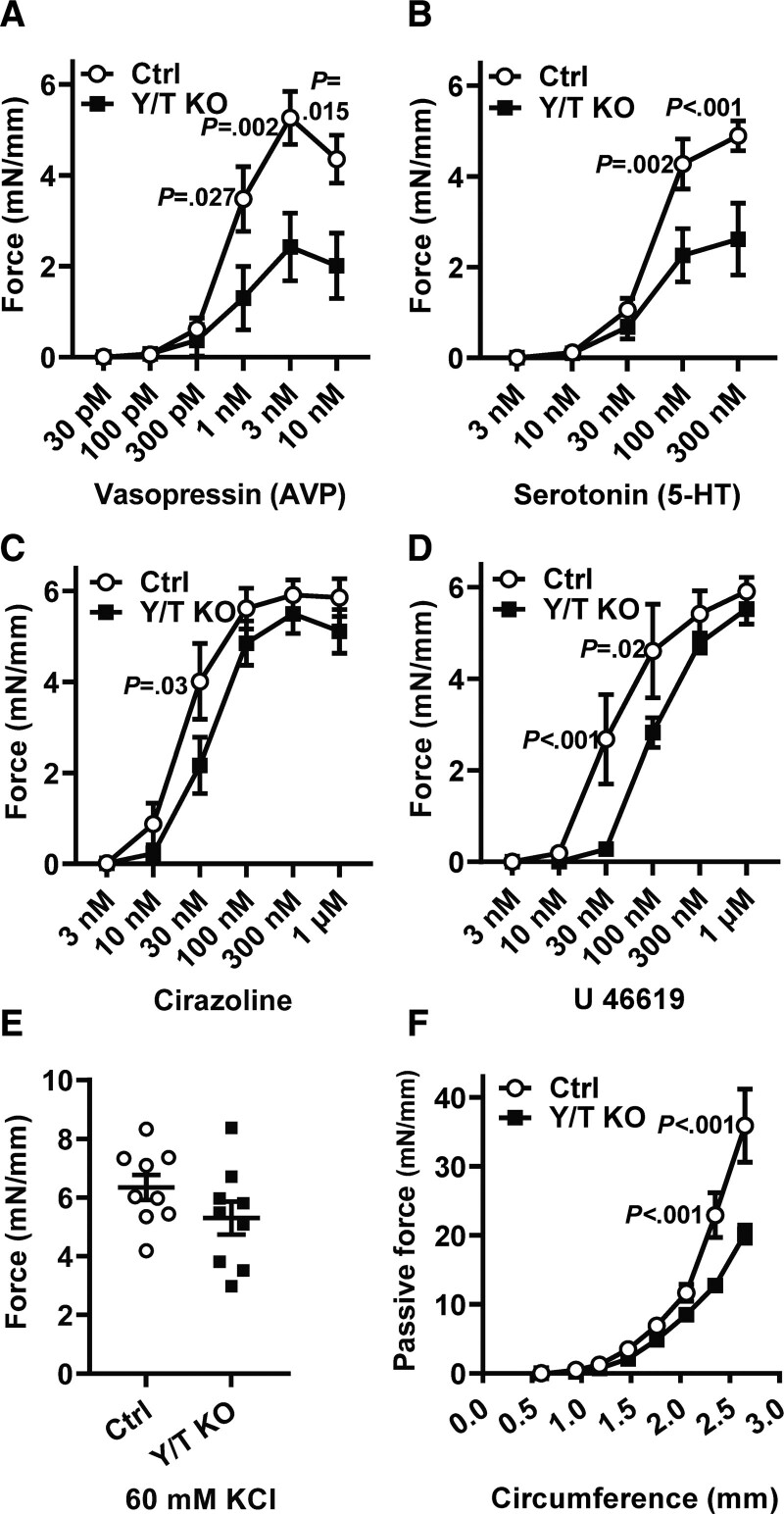
**Reduced contractile responses to various agonists and increased compliance of Y/T KO arteries.** Tail arteries were mounted in a wire myograph and dose-response curves were generated by integrating force over 7 min for each dose of (**A**) AVP (vasopressin; Ctrl [control], n=7; Y/T KO, n=8), (**B**) 5-HT (serotonin; Ctrl, n=9; Y/T KO, n=9), (**C**) cirazoline, an α_1_-adrenergic agonist (Ctrl, n=9; Y/T KO, n=9), and (**D**) U 46619, a thromboxane A2 analog (Ctrl, n=6; Y/T KO, n=6). **E**, Force development over 7-min stimulation with 60 mmol/L KCl (Ctrl, n=9; Y/T KO, n=9). **F**, The length-tension relationship of the abdominal aorta was determined in Ca^2+^-free HEPES-buffered solution (Ctrl, n=10; Y/T KO, n=8; Ctrl:vehicle-treated mice). All force measurements were normalized to the length of each preparation. All data are presented as mean±SEM. 5-HT indicates 5-hydroxytryptamine/ serotonin.

To determine the importance of YAP/TAZ for contractile function of small arteries, we first used caudal arteries mounted in wire myographs to generate dose-response curves for the vasoactive agonists AVP, serotonin, cirazoline, and U46619. The contractile force was significantly reduced at one or several concentrations of all 4 agonists in Y/T KO mice (Figure 2A through 2D). The reduction was most pronounced in the Y/T KO after stimulation with AVP and serotonin (Figure 2A and 2B). Interestingly, there was no significant difference in the response to KCl-induced depolarization between control and Y/T KO arteries 11 days after the first tamoxifen injection (Figure 2E). Although we cannot exclude a possible effect on KCl-induced contraction at later time points, the contractile responses elicited through G-protein–coupled receptors appear to be more sensitive to YAP/TAZ deletion than those elicited by membrane depolarization.

A change in the passive circumference-tension relationship in arteries is an indicator of changes in the ECM (extracellular matrix) or cytoskeletal remodeling.^22^ YAP and TAZ are known regulators of both cytoskeletal and ECM-related genes.^23^ To determine whether YAP/TAZ deletion affected passive length-tension relationship, we subjected abdominal aortae from Y/T KO and control mice to gradual increases in the internal circumference, while the resultant passive force was measured in Ca^2+^-free buffer. The passive length-tension curve of Y/T KO aortae displayed a significant decrease in tension at circumferences >2.3 mm (Figure 2F). According to the law of Laplace, the increased passive diameter of Y/T KO arteries would contribute to increased wall stress compared with control at an equal pressure level.

### Reduced Stretch-Induced Smooth Muscle–Specific Gene Expression in Y/T KO Portal Veins

Vascular mechanotransduction allows smooth muscle cells to adapt to increased mechanical stress. This adaption can be both acute, resulting in a myogenic response, or chronic, resulting in altered transcriptional activity and increased expression of contractile smooth muscle–specific genes. Determining long-term adaption to isolated mechanical stimuli in small mouse arteries is rarely performed due to technical challenges. As a surrogate, we have developed a model where portal veins are subjected to mechanical stretch by hanging a gold weight that corresponds to the optimal load for force development at one end of the vessel. This stretch is important to preserve the contractile phenotype and smooth muscle marker expression in the organ culture environment.^24,25^ Stretch-induced contractile differentiation has primarily been attributed to activation of L-type calcium influx, actin polymerization, and the actin-sensitive transcriptional coactivator MRTF.^24,26–29^ Similar to MRTF, activation of YAP/TAZ is regulated by mechanical stimuli and actin polymerization,^6^ suggesting a potential role for YAP/TAZ in the response to stretch.

**Figure 3.**
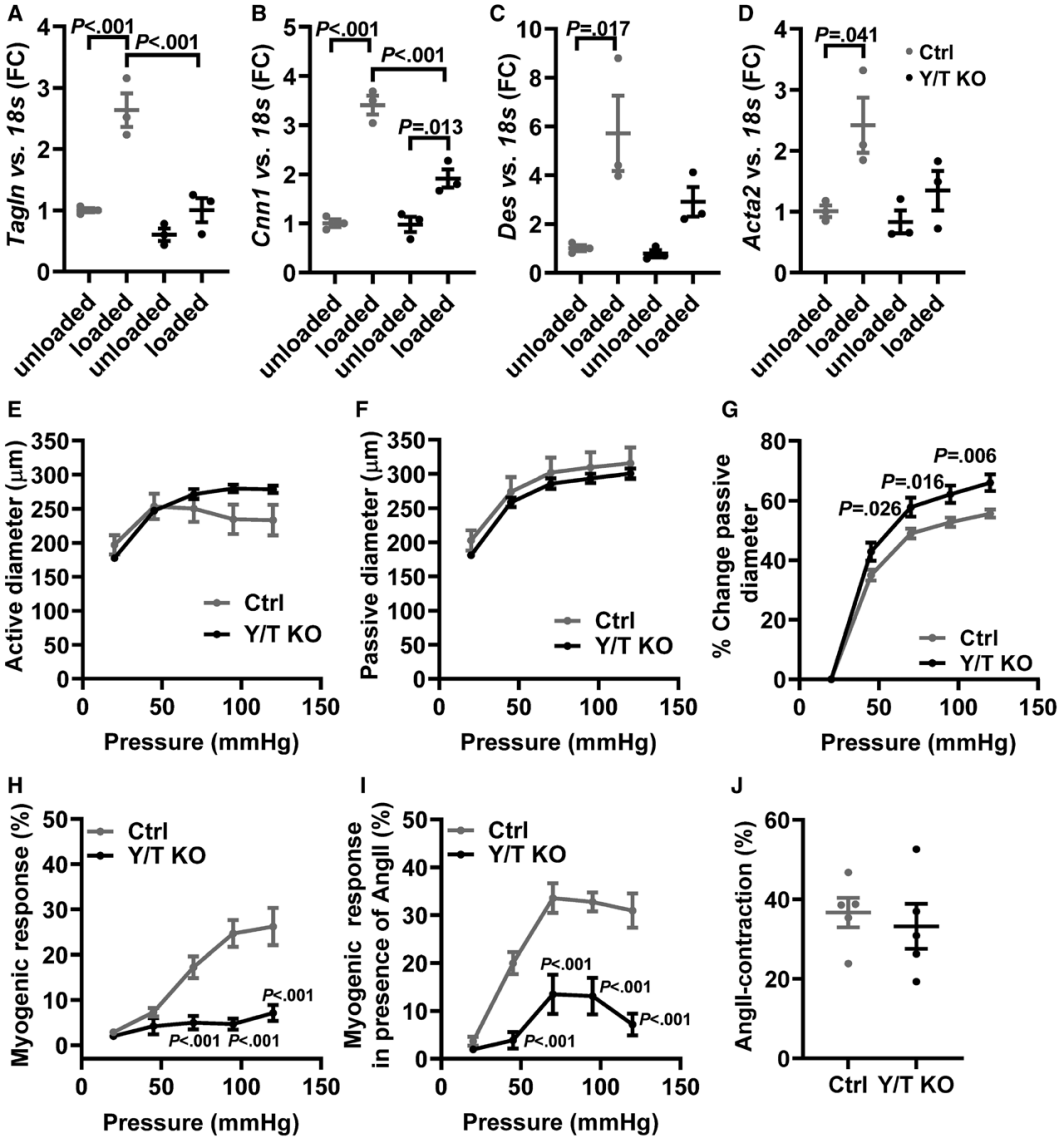
**Reduced expression of stretch-induced contractile markers and decreased myogenic response in Y/T KO (YAP/TAZ [yes-associated protein 1/WW domain containing transcription regulator 1] knockout) mice.** The portal veins (n=3 in all groups and targets) were kept in organ culture for 24 h either stretched with gold weight (loaded) or not stretched (unloaded). Reverse transcription-quantitative polymerase chain reaction analysis of selected smooth muscle markers in portal veins from Y/T KO and Ctrl (control) mice: (**A**) *Tagln*, (**B**) *Cnn1*, (**C**) *Des*, and (**D**) *Acta2*. **E–J**, Mesenteric arteries were mounted in a pressure myograph. **E**, Intraluminal pressure was increased systematically, and active vessel diameter was recorded in Ca^2+^ containing HEPES-buffered Krebs solution. **F**, Passive vessel diameter was measured in Ca^2+^-free solutions. **G**, The relative change in passive diameter was used as an indicator of vascular distensibility. **H**, Myogenic response was calculated as the relative difference between active and passive diameter. **I**, A single dose of 100 nmol/L Ang II (angiotensin II) was added to the preparations followed by gradual increase in intraluminal pressure. Active diameter was monitored, and myogenic response was calculated (**E–I**; Ctrl, n=6; Y/T KO, n=6). **J**, Peak transient contraction relative to baseline after 100 nmol/L Ang II stimulation (Ctrl, n=5; Y/T KO, n=5). All data are presented as mean±SEM. FC indicates fold change.

Portal veins were incubated either stretched or unstretched for 24 hours in organ culture. In control portal veins, stretch significantly increased the expression of the contractile smooth muscle markers: *Tagln* (transgelin/SM22α), *Cnn1*, *Des*, and *Acta2* (Figure 3A through 3D). In portal veins from Y/T KO mice, stretch-induced expression of contractile markers was blunted and only reached statistical significance for *Cnn1*. However, the expression of *Cnn1* in stretched Y/T KO portal veins was significantly lower than in the stretched control. These results suggest a role of YAP/TAZ for mechanotransduction and long-term vascular adaption to mechanical forces.

### Decreased Myogenic Response in Mesenteric Arteries of Y/T KO Mice

The myogenic response is essential for acute adaption of the vascular wall to the changes in blood pressure, and it depends on multiple factors including stretch-sensitive receptors and ion channels, Rho-signaling, and altered actin dynamics.^30–32^ To test the hypothesis that myogenic reactivity also depends on YAP/TAZ activity, small mesenteric arteries of control and Y/T KO mice were cannulated on glass pipettes and subjected to pressure myography. In this setup, active vessel diameter is continuously recorded while the intraluminal pressure is controlled using a pressure servo. Active and passive diameters were analyzed in the presence and absence of Ca^2+^, respectively (Figure 3E and 3F). In accordance with results from the aorta (Figure 2F), we found an increased distensibility of Y/T KO mesenteric arteries compared with control vessels (Figure 3G). At intraluminal pressures above 45 mm Hg, control arteries develop pressure-dependent myogenic response (Figure 3H). However, in arteries deficient in YAP/TAZ, the myogenic responses were essentially absent at all pressure levels (Figure 3H). Angiotensin II receptors are known to be involved in the myogenic response,^30^ and we have previously demonstrated that angiotensin II stimulation can rescue the loss of myogenic tone in other KO models.^33^ In mouse mesenteric arteries, angiotensin II stimulation (100 nmol/L) normally results in a transient contraction that returns to baseline after ≈10 minutes. Myogenic responses were then evaluated in the presence of angiotensin II and found to be potentiated in control arteries (Figure 3I). Although Y/T KO arteries also developed moderate myogenic tone following angiotensin II stimulation, the difference between control and KO arteries increased further and was also significantly different at 45 mm Hg (Figure 3I). The acute contractile response to angiotensin II was similar in control and Y/T KO vessels, but this was only determined for one concentration (100 nmol/L; Figure 3J). Taken together, these results demonstrate an essential role of YAP/TAZ for the myogenic response in small arteries.

### YAP/TAZ Regulates Genes Involved in Vascular Smooth Muscle Differentiation and Contractile Function

**Figure 4.**
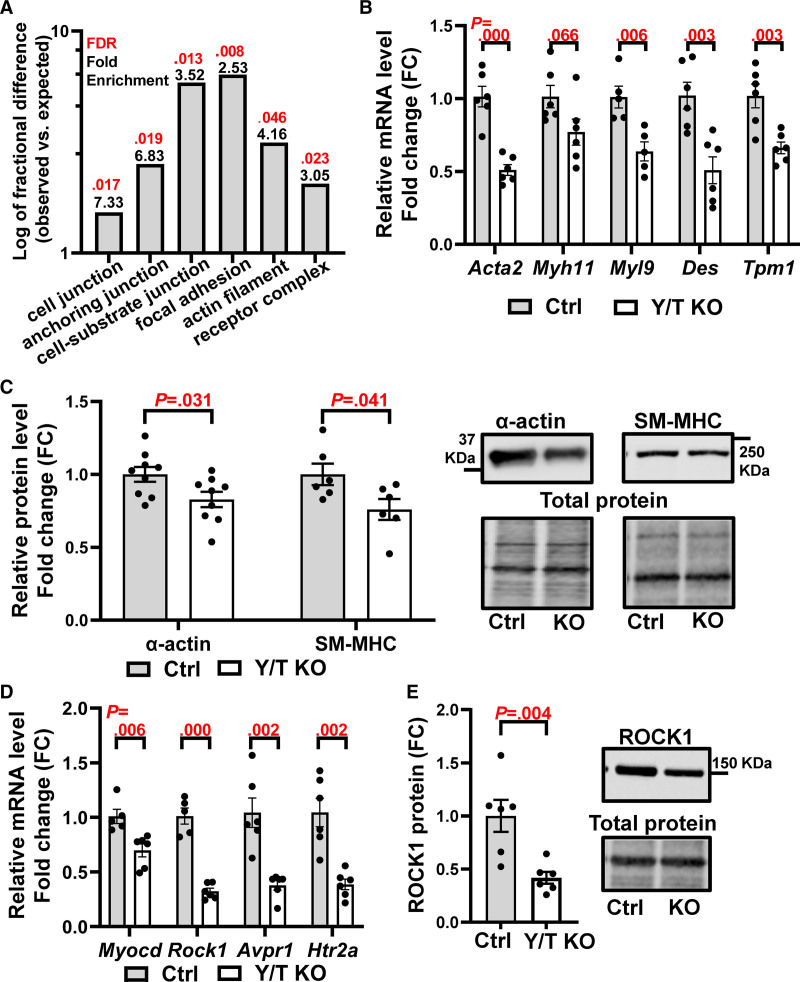
**Downregulation of genes involved in vascular smooth muscle differentiation and contraction in Y/T KO (YAP/TAZ [yes-associated protein 1/WW domain containing transcription regulator 1] knockout) mice.** RNA sequencing was performed on aortae from Ctrl (control) and Y/T KO mice. **A**, Protein Analysis Through Evolutionary Relationships (PANTHER) overrepresentation test and PANTHER GO-slim cellular component of significantly downregulated genes with fold enrichment in black and false discovery rate in red. **B**, Confirmation of selected smooth muscle markers by RT-qPCR (Ctrl, n=5–6; Y/T KO, n=5–6). **C**, Quantification of Western blot analysis of SM-MHC (smooth muscle α-actin and myosin heavy chain) and their representative blots to the right. Targets were normalized to the total protein (Ctrl, n=6–9; Y/T KO, n=6–9). **D**, Reverse transcription-quantitative polymerase chain reaction analysis of *Myocd*, *Rock1*, *Avpr1*, and *Htr2a* mRNA expression (Ctrl, n=5–6; Y/T KO, n=6). **E**, Western blot analysis of ROCK1 (Rho kinase 1; Ctrl, n=6; Y/T KO, n=6). All data are presented as mean±SEM. FC indicates fold change; FDR, false discovery rate; and KO, knockout.

YAP and TAZ are transcriptional regulators affecting genes involved in growth, metabolism, ECM production, and cytoskeletal dynamics.^6,34^ The specific repertoire of YAP/TAZ-regulated genes is likely dependent on the cell type, tissue, and environmental cues. We previously reported the transcriptomic impact of Y/T KO in bladder and colon.^13^ To gain further insights into the molecular mechanisms involved in the effects of YAP/TAZ deletion in the vasculature, we performed RNA-seq using control and Y/T KO aortae. GO enrichment analysis of the significantly downregulated genes demonstrated the involvement of focal adhesion (*Itgb8*, *Itgbl1*, and *Lpp*), cell junction (*Cdh11*, *Cdh3*, and *Gpc6*), actin filaments (*Tpm1*, *Cobl*, and *Dbn1*), and receptor complex genes (*Plxna4*, *Ramp1*, and *Epha1*; Figure 4A; Table S1). RNA-seq also revealed downregulation of several smooth muscle markers, and this was confirmed by either reverse transcription-quantitative polymerase chain reaction or Western blotting (Figure 4B and 4C). The selection of the confirmed genes was based on published results describing important modulators of smooth muscle contraction. For example, *Myocd* is a potent transcriptional coactivator controlling smooth muscle differentiation by binding to SRF (serum response factor).^35,36^ Myocardin expression was significantly reduced in Y/T KO (Figure 4D), whereas the expression of SRF was unchanged both at the mRNA and protein levels (Figure S1A and S1B). In addition to myocardin, ROCK1, which plays a role in Ca^2+^ sensitization and smooth muscle contraction,^37^ was downregulated both at the mRNA and protein level in Y/T KO aortae (Figure 4D and 4E). Reduced expression of the AVP V1a receptor (*Avpr1a*) and serotonin receptor 2A (*Htr2a*) was confirmed by reverse transcription-quantitative polymerase chain reaction (Figure 4D). These latter findings provide additional explanations for the reduced contractile responses to AVP and serotonin, respectively.

**Figure 5.**
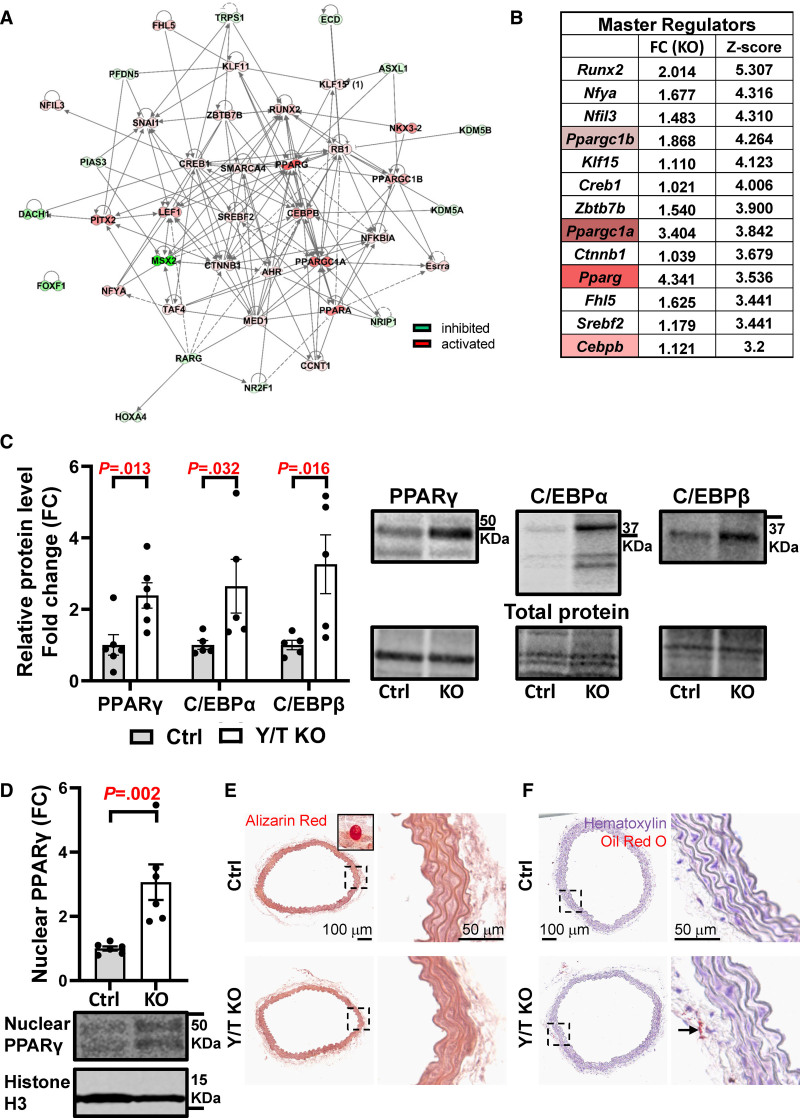
**Upregulation of transcription factors that are involved in adipogenic differentiation: PPARγ (peroxisome proliferator activated receptor gamma), C/EBPα (CCAAT enhancer binding protein alpha), and C/EBPβ (CCAAT enhancer binding protein beta). A**, Network of the predicted master regulators that are involved in transcription regulation. The interactions between the nodes are based on Qiagen ingenuity knowledge base, where the solid and dashed lines represent direct and indirect interaction, respectively. The intensity of coloring is based on the magnitude of *Z* score values ranging from strong inhibition (dark green) to strong activation (dark red). **B**, The top 13 master regulators and their fold change and *Z* score, which reflects their activation status. **C**, Quantification of Western blot analysis of PPARγ, C/EPBα, and C/EPBβ and representative blots to the right (Ctrl [control], n=5–6; Y/T KO [YAP/TAZ (yes-associated protein 1/WW domain containing transcription regulator 1) knockout], n=5–6). **D**, Quantification and representative blot showing PPARγ in the nuclear fraction and histone H3 as nuclear marker (Ctrl, n=6; Y/T KO, n=6). **E**, Alizarin Red staining for calcium deposits detection in aortae of Ctrl and Y/T KO mice. Inset represents positive Ctrl (bone). **F**, Assessment of lipid accumulation in the aorta by Oil Red O staining, positive staining in perivascular fat (arrow). Detailed legend description for IPA network is available online: https://qiagen.secure.force.com/KnowledgeBase/articles/Knowledge/Legend. All data are presented as mean±SEM. Ctrl indicates control; DAPI, 4′,6-diamidino-2-phenylindole; FC, fold change; and KO, knockout.

To investigate the influence of YAP/TAZ deletion on transcriptional regulation, prediction of master regulators was performed using the Ingenuity Pathway Analysis software, based on significantly regulated genes in the RNA-seq data set. Positive or negative *Z* scores were given to the master regulators to reflect their activation or inhibition, respectively. Selection of master regulators was performed to include genes involved with an absolute *Z* score above 2 and matched direction of fold change and *Z* score. This selection resulted in 41 master regulators represented in the interactome (Figure 5A). After ranking the master regulators by their *Z* score and fold change, a top list of 13 activated regulators was obtained (Figure 5B). Interestingly, beyond *Runx2*, *Pparg* and its coactivators *Ppargc1a* and *Ppargc1b* were among the top master regulators. In addition to a well-documented role in adipocyte differentiation,^38^ PPARγ has also been identified as a potent antagonist of contractile smooth muscle differentiation.^39^ We, therefore, examined the PPARγ level by Western blotting and corroborated an upregulation in Y/T KO aorta (Figure 5C). An increased expression of PPARγ could potentially stimulate adipogenic differentiation of smooth muscle cells, and, indeed, a number of adipogenic markers were upregulated in Y/T KO aorta (Figure S1C). Interestingly, C/EBPβ, known to induce adipocyte differentiation, was also among the top 13 master regulators. Both C/EBPβ and C/EBPα were significantly upregulated at the protein level (Figure 5C). Moreover, increased expression of PPARγ in Y/T KO was also observed in the nuclear fraction, further supporting PPARγ activation (Figure 5D). Representative blots of cytoplasmic and nuclear fractions are depicted in (Figure S1E).

In accordance with Wang et al,^14^ we found a significant increase in genes regulating osteogenic differentiation in Y/T KO mice (Figure S1D). To test whether these transcriptional changes resulted in increased calcification or lipid accumulation, we stained aortic sections with Alizarin Red S and Oil Red O, respectively (Figure 5E and 5F). However, these stainings were negative in the media of both control and Y/T KO aorta at 9 to 11 days after the first tamoxifen injection.

Having demonstrated that knockout of YAP and TAZ causes reduction of myocardin and smooth muscle markers in mouse arteries, together with a reciprocal increase in PPARγ, we next investigated whether YAP and TAZ play a similar role for regulation of these transcripts in human arteries. For this, we downloaded RNA-seq data from GTEXPortal.org and performed correlation analyses (Spearman). We observed that *YAP1* correlated tightly and consistently across arteries with *MYOCD*, *ROCK1*, and smooth muscle markers as predicted. The only exception from the pattern observed in mouse was that *AVPR1A* failed to correlate positively with *YAP1* throughout (Figure S2A through S2C). TAZ (*WWTR1*) showed a similar correlation pattern, especially in the coronary and tibial arteries (second row/column in the heat map, individual correlations are not shown).

### Rapid Development of Vascular Lesions in Hypertensive Y/T KO Mice

**Figure 6.**
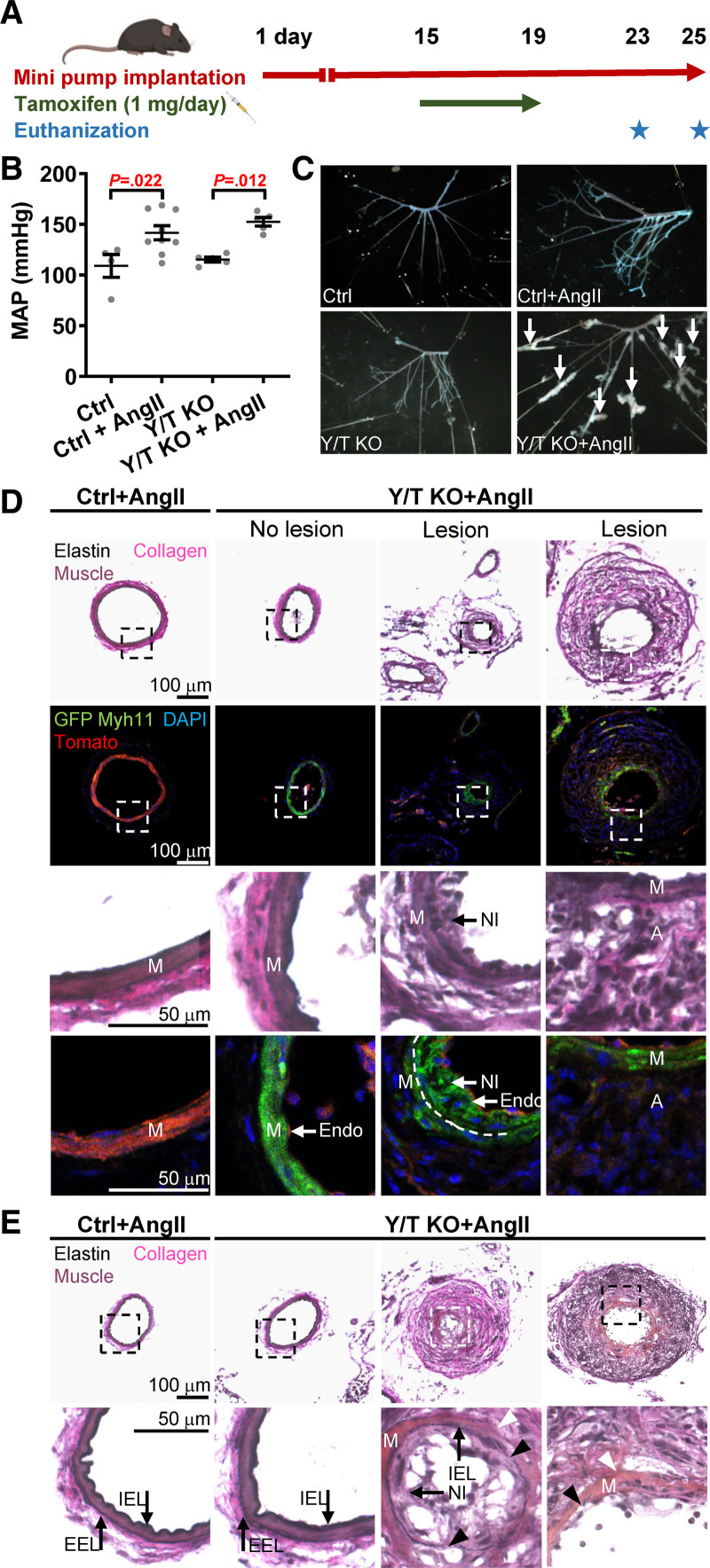
**Vascular lesion development in hypertensive Y/T KO (YAP/TAZ [yes-associated protein 1/WW domain containing transcription regulator 1] knockout) mice. A**, Timeline representation of osmotic pump implantation and knockout induction. **B**, Blood pressure measurement just before euthanization of Ctrl (control) and Y/T KO mice using the tail cuff method. **C**, Representative images of mesenteric arteries from Ctrl and Y/T KO mice after removal of surrounding fat and tissue. Lesions are highlighted with arrows. **D**, Bright-field and fluorescence images of cryosections of mesenteric arteries isolated from Ctrl (YAP^fl/fl^/TAZ^fl/fl^ Cre-negative ROSA^mT/mG^) and Y/T KO Cre reporter mice (YAP^fl/fl^/TAZ^fl/fl^ Cre/ERT2 ROSA^mT/mG^) at 9 or 11 d after the first tamoxifen injection. Top and third row: Verhoeff Van Gieson staining to visualize vascular lesions and the different layers of the vessel wall. Second and fourth row: fluorescence images demonstrating Cre recombination event by using ROSA ^mT/mG^ Cre reporter mice. Upon tamoxifen-induced activation of *Myh11*-Cre, the Tomato transgene (red) is excised and replaced by the expression of GFP (green fluorescence protein) in smooth muscle. Note that many of the cells of the neointima are positive for GFP, whereas the cells of the thickened adventitia and endothelial cells are negative. DAPI (4′,6-diamidino-2-phenylindole) was used as a nuclear stain. Dashed line represents the internal elastic lamina. **E**, Verhoeff Van Gieson staining of mesenteric arteries of Y/T KO mice demonstrates sites of degradation of both the external (white arrowheads) and internal (black arrowheads) elastic lamina (**D** and **E**; Ctrl+Ang II [angiotensin II], n=3; Y/T KO+Ang II, n=3). All data are presented as mean±SEM. A indicates adventitia; EEL, external elastic lamina; Endo, endothelium; IEL, internal elastic lamina; M, media; MAP, mean arterial pressure; Myh11‚ myosin heavy chain 11; and NI, neointimal.

Our results suggest that loss of YAP/TAZ limits the ability of the vascular smooth muscle to adapt to mechanical forces, both acutely and chronically. To examine the impact of impaired mechanotransduction in vivo, angiotensin II minipumps were implanted in the mice 2 weeks before tamoxifen injection. The mice were euthanized 9 to 11 days after the first tamoxifen injection (Figure 6A). Y/T KO in the setting of established hypertension did not affect blood pressure (Figure 6B). Analysis of microdissected mesenteric arterial trees revealed development of vascular lesions in hypertensive Y/T KO mice (Figure 6C). Multiple lesions were observed in all angiotensin II–treated Y/T KO mice (n=8). In control mice, only a single lesion in one angiotensin II–treated control mouse (n=9) was detected. No lesions were observed in the mesenteric arteries of normotensive control or Y/T KO mice. Furthermore, we did not observe any morphological differences between aortae from control and Y/T KO mice, regardless of normotensive or hypertensive conditions. However, both control and Y/T KO aorta exhibited modest structural changes in hypertensive compared with normotensive conditions (Figure S3A).

The vascular lesions exhibited both adventitial and neointimal hyperplasia (Figure 6D). To determine the contribution of smooth muscle cells to the neointimal and adventitial hyperplasia, smooth muscle lineage-tracing experiments were performed using ROSA^mT/mG^ reporter mice. YAP^fl/fl^/TAZ^fl/fl^ Cre-negative ROSA^mT/mG^ mice were used as controls. Both ROSA^mT/mG^ Y/T KO and controls were subjected to angiotensin II via osmotic minipumps as described above. In this lineage-tracing model, all cells exhibit red emission before Cre recombination. Cre recombination in mice without the floxed genes was not evaluated herein, but the smooth muscle specificity of *Myh11*Cre activity has been demonstrated previously.^16,40^ After tamoxifen injection, only the cells that express *Myh11* replace the red fluorescence with green fluorescence. Interestingly, neointimal cells were strongly positive for GFP (green fluorescence protein), suggesting that these cells originated from smooth muscle cells (Figure 6D). However, cells of the thickened adventitia were predominantly negative for GFP, indicating that these cells are likely not derived from smooth muscle cells (Figure 6D). Since the mice were subjected to angiotensin II 2 weeks before tamoxifen treatment, we cannot exclude that some of the red cells outside of the medial layer are derived from smooth muscle cells. However, green cells can only be derived from smooth muscle cells that expressed *Myh11* at the time of tamoxifen administration. Moreover, loss of YAP and TAZ resulted in severe degradation of both internal and external elastic laminae in mesenteric arteries (Figure 6E).

**Figure 7.**
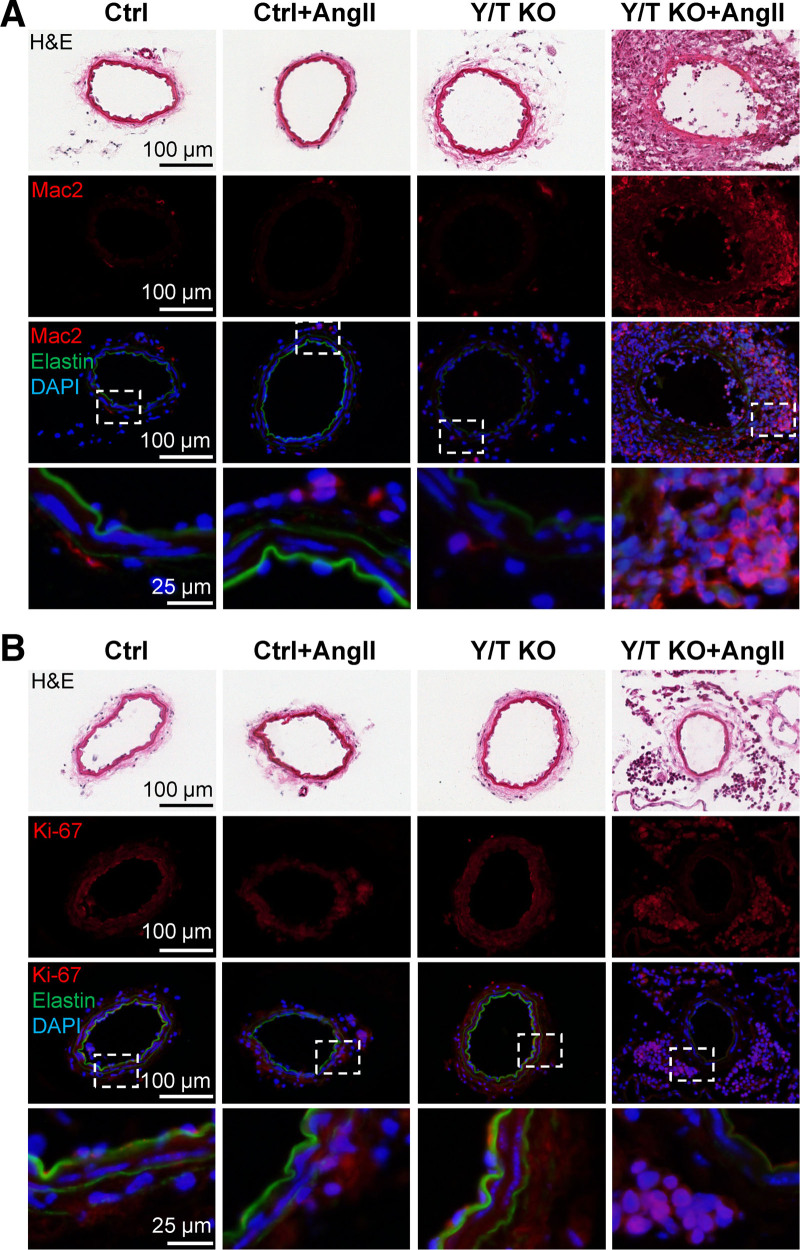
**Marked macrophage infiltration and increased rate of proliferation in the adventitia of the vascular lesions.** Cryosections of mesenteric arteries from Ctrl (control) or Y/T KO (YAP/TAZ [yes-associated protein 1/WW domain containing transcription regulator 1] knockout) mice treated with Ang II (angiotensin II) or vehicle. **A**, Immunostaining for Mac2 (galectin 3) demonstrates pronounced infiltration of monocytes/macrophages in the adventitia of hypertensive Y/T KO vascular lesions. **B**, The thickened adventitia of Y/T KO vascular lesions is associated with intense cell proliferation (Ki-67), whereas the media displays no obvious signs of cell proliferation. DAPI (4′,6-diamidino-2-phenylindole) was used as a nuclear stain. Elastin autofluorescence is shown in green. H&E indicates hematoxylin and eosin.

To further characterize the vascular lesions, sections of mesenteric arteries were stained for Mac2, a macrophage marker, and Ki-67, a cell proliferation marker. The adventitia within the lesions was heavily infiltrated with Mac2 positive cells and cells that stained strongly for Ki-67 (Figure 7A and 7B). Nonmerged images for all fluorophores are shown in (Figure S3B and S3C).

### Angiotensin II–Induced Hypertension Further Represses Vascular Contractility in Y/T KO Mice

To determine the effect of angiotensin II–induced hypertension on smooth muscle contractility in control and Y/T KO mice, we performed wire myography experiments on tail arteries from control and Y/T KO mice, subjected to 4 weeks of angiotensin II–induced hypertension. The contractile function of angiotensin II–treated mice was compared with untreated mice from the previous results (Figure 2, the black curves). Y/T KO arteries displayed further reduction in agonist-induced contractile force to AVP and serotonin and a comparable reduction to cirazoline, whereas control mice only demonstrated a significant reduction of AVP-induced contraction (Figure S4A through S4F). We did not test contractile responses to U46619 in hypertensive mice. Taken together, these results argue that angiotensin II–induced hypertension accentuates the reduced contractility in Y/T KO arteries.

## Discussion

In this study, we demonstrate that YAP and TAZ are critical for maintaining vascular wall integrity and function and that YAP and TAZ play a pivotal role for reducing the risk of hypertension-induced vascular diseases in mice. Deletion of YAP/TAZ in smooth muscle cells alters the transcriptional program toward a less contractile phenotype. Functionally, blood vessels depleted of YAP/TAZ exhibit reduced agonist-induced contractility, loss of pressure-induced myogenic responses, and aberrant stretch-induced gene expression. These defects are likely contributing to the reduced ability of Y/T KO vessels to adapt to increased systemic blood pressure‚ hence lead to hypertension-induced vascular lesions development.

Hypertension is a major risk factor for cardiovascular diseases, and it is, therefore, of crucial importance to identify why and how increased blood pressure can damage the vascular wall. According to the law of Laplace, wall stress is increased by elevated transmural pressure, an increase in vessel radius, or a decrease in wall thickness. Therefore, small resistance arteries can normalize wall stress following an increase in systemic blood pressure by several protective mechanisms: (1) the myogenic response acutely reduces vessel diameter within minutes after an increased wall stress has been detected by the vascular smooth muscle cells.^1^ (2) Prolonged agonist-induced constriction can cause inward remodeling resulting in a permanent reduction of vessel diameter and increased media/lumen ratio.^41^ Inward remodeling has also been observed in patients with essential hypertension.^42^ (3) Following >24 hours of increased wall stress, vascular smooth muscle cells start to produce increased amounts of contractile and cytoskeletal proteins.^25,26,28,41^ These adaptive mechanisms aim to reduce the harmful effects of increased wall stress, and, interestingly, all of them are impaired in Y/T KO arteries.

Despite the clear-cut reduction of vascular reactivity, Y/T KO did not reduce established hypertension. This is not surprising considering (1) that hypertension had already developed before knockout induction, (2) that angiotensin II–induced contraction was similar in control and Y/T KO at 11 days, and (3) that angiotensin II–induced hypertension in mice may primarily be due to smooth muscle–independent effects, such as increased fluid retention.^17^ The resulting increase in blood pressure combined with defective adaptive mechanisms in Y/T KO mice is likely contributing to elevated wall stress and lesion development in small arteries.

Importantly, as evident by the absence of lesions in normotensive Y/T KO mice, it is not the deletion of YAP/TAZ per se that results in lesion development in this model. Similarly, we have previously reported that mice harboring a deletion of miR-143/145, which results in impairment of contractile function and mechanosensing, develop vascular lesions in a hypertensive but not normotensive setting.^17,29^ Our results thus support a general theory, suggesting that reduced expression or function of components that are essential for vascular smooth muscle mechanotransduction and adaption to increased wall stress results in an increased susceptibility to hypertensive vasculopathy. This theory is further supported by clinical data suggesting that impaired vascular contractility can cause aneurysms, for example, due to mutations in genes encoding contractile proteins.^43,44^

The lesions in Y/T KO mesenteric arteries are characterized by neointimal and adventitial hyperplasia, elastin degradation, and infiltration of inflammatory cells, all of which are characteristic hallmarks of vascular diseases, including aneurysm formation. In agreement with previous reports, we demonstrate by lineage-tracing experiments that vascular smooth muscle cells migrating from the media are the main contributors of neointimal hyperplasia.^40,45^ The low level of Ki67 staining in the media suggests that these cells were nonproliferative at the time of analysis. In contrast to our findings, it has been reported that inhibition of YAP leads to alleviation of neointimal formation after carotid ligation.^46^ However, the approach used by Wang et al^46^ in that article was not smooth muscle specific and only targeted YAP expression, which may explain the discrepancy between the studies.

Vascular lesions and repair also typically involve the adventitial layer of the vascular wall characterized by fibroblast cell proliferation and infiltration of immune cells, together resulting in a profound expansion of the adventitia.^47–49^ Accordingly, vascular lesions occurring in hypertensive Y/T KO mice displayed intense adventitial cell proliferation and contained a large number of Mac2^+^ cells, typically linked to inflammation and fibrosis.^50,51^ Our lineage-tracing data do not support any significant contribution of smooth muscle–derived cells in the development of adventitial hyperplasia. This suggests that the contribution of smooth muscle cells to vascular wall remodeling proceeds in a media-intima direction rather than a media-adventitia direction.

Considering the dramatic change in morphology of the vascular lesions, it is noteworthy that they develop already at 9 to 11 days following YAP/TAZ deletion, although the mice were exposed to angiotensin II for a longer time (3–4 weeks). The angiotensin II–treated control mice, with a single exception, did not develop lesions. The lesions thus start to develop upon deletion of YAP/TAZ. Since the complete loss of YAP/TAZ proteins in smooth muscle is expected to require a few days from the first tamoxifen injection, the lesions most likely develop within 1 week after removal of YAP/TAZ proteins in the smooth muscle, demonstrating an astonishingly rapid process.

In good agreement with our current findings, Wang et al also found that YAP and TAZ are required to maintain the contractile phenotype of smooth muscle cells utilizing the same knockout model.^14^ Correlation analyses between our and Wang et al RNA-seq data sets using the Spearman method resulted in correlation coefficient of R=0.518, *P*<0.0001, and this coefficient reached 0.902, *P*<0.0001 when only significantly regulated genes were analyzed. The two data sets, therefore, support each other. Wang et al^14^ also reported that loss of YAP/TAZ leads to osteogenic transdifferentiation of smooth muscle cells and vascular calcification. We were not able to detect vascular calcification in the present work. A possible explanation for this discrepancy is the longer time allowed between KO induction and sacrifice in the work by Wang et al (22 days) compared with our study (9–11 days). Ethical constraints prevented us from examining mice at 22 days of KO induction, but already at 9 days, Runx2—an important regulator of osteogenic differentiation—was among the master regulators of gene expression identified by analysis of differentially expressed genes in the Y/T KO aorta. Hence, we believe that our findings are consistent in this regard and that an inducible smooth muscle KO model that circumvents the gastrointestinal phenotype would shed further light on this effect.

Identification of master regulators in our RNA-seq data highlighted *Pparg* and its coactivators *Ppargc1a* and *Ppargc1b*. PPARγ is a nuclear hormone receptor and ligand-activated transcription factor that is involved in adipocyte differentiation, glucose metabolism, and inflammation.^38,52^ Although direct activation of PPARγ was not measured, increased protein expression of PPARγ was observed in nucleus of Y/T KO aortic cells. Interestingly, a mouse model of smooth muscle–specific PPARγ overexpression resembles Y/T KO in many aspects.^39^ Both PPARγ overexpression and loss of YAP and TAZ result in an attenuated contractile response, increased aortic compliance, reduced expression of smooth markers, and increased expression of adipogenic markers. Moreover, PPARγ overexpression causes downregulation of myocardin, explaining the decreased expression of smooth muscle markers. Similar findings of reduced smooth muscle markers and upregulation of adipogenic markers were also observed after overexpressing PPARγ in rat vascular smooth muscle cells in vitro.^53^ In accordance with this, Nobusue et al also demonstrated an antagonistic relationship between PPARγ and MRTFA during adipogenesis.^54^

The GO analysis suggests that pathways related to, for example, focal adhesions, actin filaments, and receptor complexes are enriched among differentially expressed genes. Myocardin and ROCK1 are well-known regulators of cytoskeletal components and focal adhesions, and PPARγ has been demonstrated to antagonize the effect of myocardin. The master regulator analysis thus suggests a reasonable hypothesis for enrichment in these GO categories (Y/T knockout→PPARγ activation→reduced myocardin activity→reduced actin filaments and focal adhesions). The receptor complex category may involve a distinct chain of events given that myocardin, to the best of our knowledge, does not control *Avpr1a* or *Htr2a*. One possibility is that these transcripts could be direct targets of YAP/TAZ-TEAD signaling, but further studies are required to test this hypothesis.

In addition to PPARγ activation, the induction of adipogenic markers could be regulated by other factors, such as C/EBPα and C/EBPβ, which play a pivotal role in adipocyte differentiation.^55,56^ These transcriptional factors can work independently and synergistically with PPARγ.^57–59^ It has been reported that PPARγ promotes lipid accumulation in the vascular wall after 4 weeks of overexpression.^39^ However, we did not observe an increased lipid deposition in Y/T KO arteries. This again could be attributed to the short life span of Y/T KO mice.

Expression of YAP/TAZ in human disease was not analyzed in this study. However, we demonstrate highly significant relationships between *YAP1* and *WWTR1* with smooth muscle–specific gene expression in human arteries, suggesting an important regulatory role of YAP and TAZ for expression of these transcripts in the human vasculature.

Taken together, we show in several vascular beds that YAP and TAZ are important for regulating mechanisms involved in the adaption of the vascular wall to increased mechanical stress. Importantly, we also demonstrate that small resistance arteries deficient of YAP/TAZ become vulnerable to a remarkably rapid development of hypertension-driven vascular lesions. The lesions in Y/T KO mesenteric arteries exhibited several hallmarks of vascular diseases in humans suggesting that the proposed mechanisms are likely to be relevant for human vascular diseases. These mechanoresponsive coactivators and their upstream regulators are thus promising targets for novel therapies to combat vascular disease.

## Article Information

### Acknowledgments

We thank the Center for Translational Genomics, Lund University and Clinical Genomics Lund, SciLifeLab for providing RNA sequencing service. The authors also thank Katarzyna Kawka for technical assistance and Dr Karin Stenkula for providing C/EBP antibodies. Graphic abstract was created with Biorender.com.

### Sources of Funding

This work was supported by grants from the Novo Nordisk Foundation (SA: 34366), the Swedish Research Council (SA: 2017–00860, 2020-01145, KS: 2020-00908), the Swedish Heart and Lung Foundation (SA: 20200322, KS: 20200222), The Crafoord Foundation, The Magnus Bergvall Foundation, the Royal Physiographic Society, the Lars Hierta Foundation, and the Greta and Johan Kock Foundation. F. Daoud was supported by a stipend from the University of Jordan.

### Disclosures

None.

### Supplemental Material

Figures S1–S4

Table S1

## Supplementary Material


