# Supplementary material for: YAP and TAZ in Vascular Smooth Muscle Confer Protection Against Hypertensive Vasculopathy
Source: Arterioscler Thromb Vasc Biol. 2022 Feb 24;42(4):428–43. doi: 10.1161/ATVBAHA.121.317365 (PMC8939708; doi:10.1161/ATVBAHA.121.317365)
Supplement: Supplementary file 1 [file atv-42-428-s001.pdf]

"SUPPLEMENTAL MATERIALS"

YAP and TAZ in vascular smooth muscle confer protection against hypertensive vasculopathy

Fatima Daoud[1], Marycarmen Arévalo Martinez[1], Johan Holmberg[1], Azra Alajbegovic[1], Neserin Ali[2], Catarina Rippe[1], Karl Swärd[1] and Sebastian Albinsson[1*]

[1]Department of Experimental Medical Science, Lund University, Sweden.
[2]Department of Clinical Sciences Lund, Orthopaedics, Clinical Epidemiology Unit, Lund University, Sweden

*Corresponding author:
Department of Experimental Medical Science, Lund University, BMC D12, SE-221 84 Lund, Sweden.
E-mail address: sebastian.albinsson@med.lu.se.
Phone: +46-46-2220678

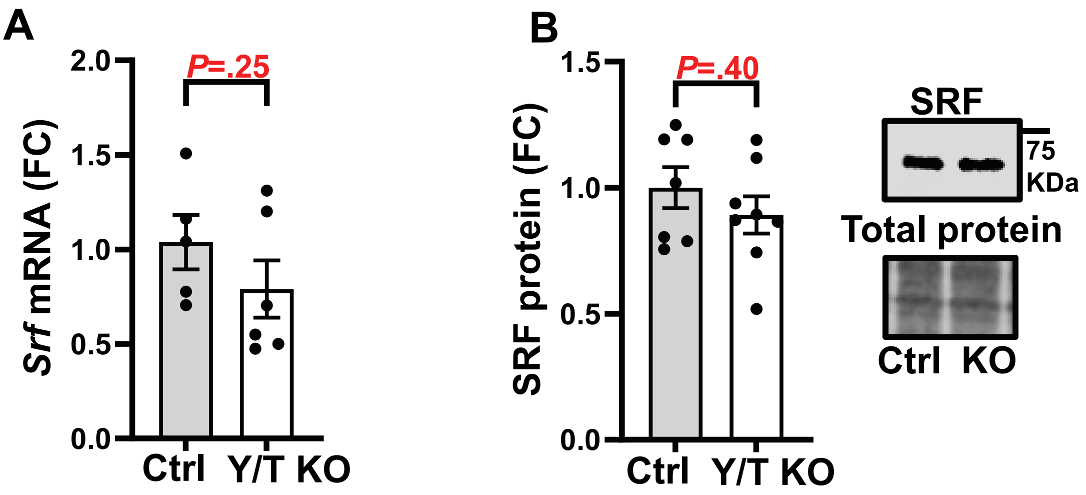

C

Adipogenic markers		
**Gene**	**FC (KO)**	***P* adj**
Adipoq	6.090	0.052
Adipor2	1.636	0.011
Cd36	3.422	0.081
Cfd	6.163	0.080
Cidea	4.093	0.006
Fabp4	3.802	0.010
Lep	6.245	0.064
Plin2	1.811	0.024
Retn	4.791	0.073
Slc27a2	14.140	0.089

D

Osteogenic markers		
**Gene**	**FC (KO)**	***P* adj**
Acan	5.729	1.63E-16
Alpl	1.638	0.395
Bmp2	1.811	0.036
Bmp4	1.882	1.12E-08
Col1a2	0.822	0.489
Mgp	1.670	0.001
Runx2	2.014	NA
Sox9	3.756	1.01E-07
Spp1	3.980	0.002
Tnfrsf11b	1.646	0.102
Vcan	2.244	8.99E-07

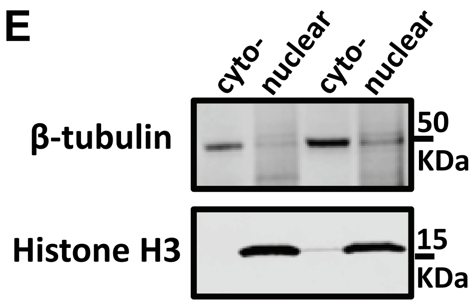

Figure S1. Expression of selected transcripts.
(A) *Srf* mRNA level normalized to *Gapdh.* (B) Quantification of western blot analysis of SRF to the left and a representative blot to the right. Protein level was normalized to total protein stain. (C) Selection of some adipogenic markers from RNA sequencing data with their fold changes in Y/T KO (FC KO) and adjusted P value. (D) Selection of some osteogenic markers from RNA sequencing data with their fold changes in Y/T KO (FC KO) and adjusted P value. (E) representative blot of nuclear and cytoplasm separation; β- tubulin as cytoplasmic marker and Histone H3 as nuclear marker.

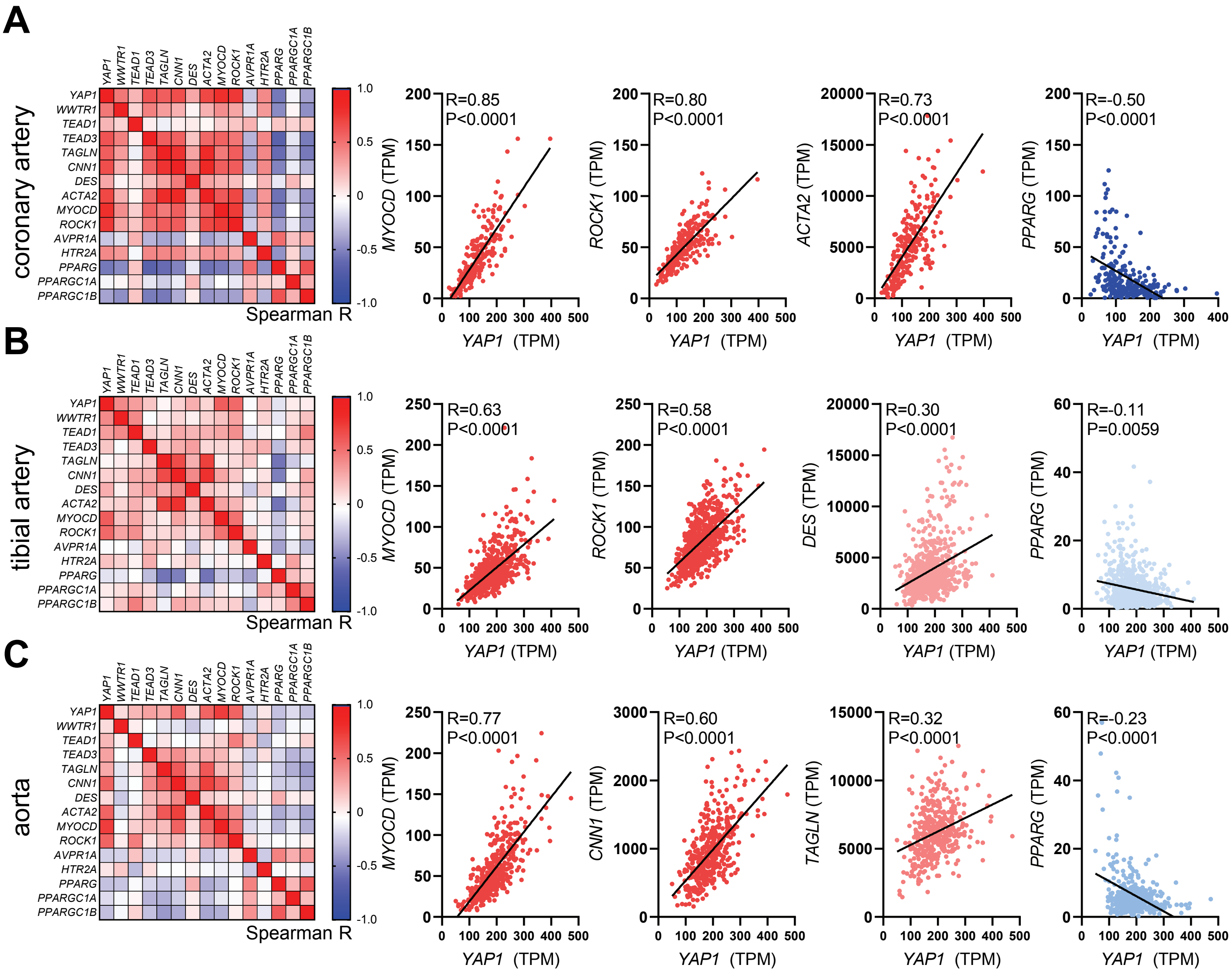

Figure S2. Similar relationships between *YAP1* and *WWTR1* (TAZ) and smooth muscle markers in human and mouse arteries. Downloaded RNA-sequencing data from the GTEXPortal.org followed by correlation analyses. The heatmaps to the left (A-C) are visual representation of the Spearman R-value that resulted from different correlations, ranging from -1 (blue) to +1 (red). Each matrix represents correlation between *YAP1* (First row/column), *WWTR1* (second row/column), and several other transcripts that we identified in the mouse in different vascular beds (A) coronary artery (B) tibial artery (C) aorta. To the right of each matrix, *YAP1* exemplary correlations for human coronary artery (*n*=240), tibial artery (*n*=663), and aorta (*n*=432) are shown. P-values and Spearman R coefficients are specified in the latter panels. TPM: transcripts per million.

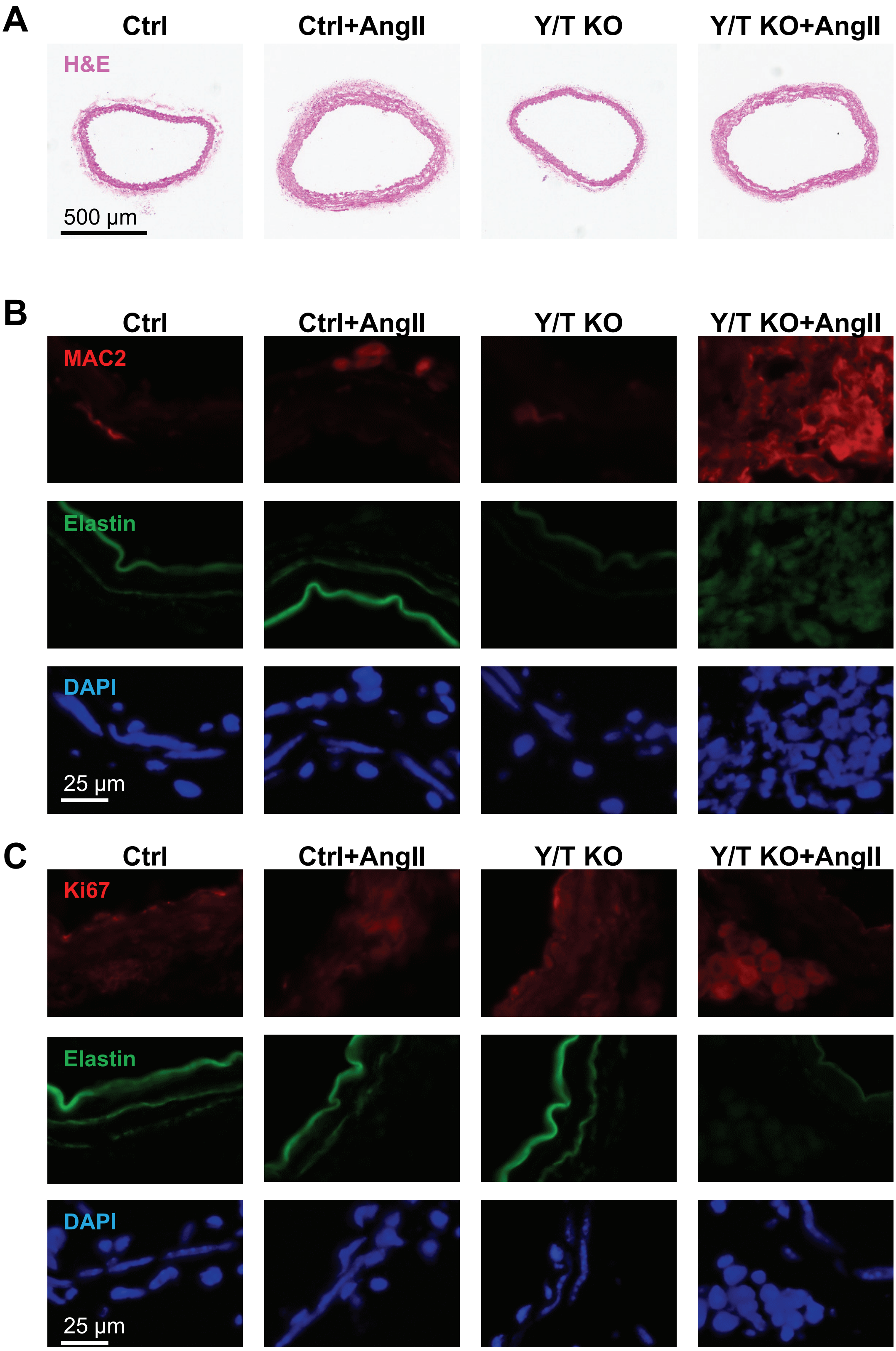

Figure S3. Histological images of aortae and fluorescence images of mesenteric arteries. (A) Hematoxylin and eosin staining (H&E) of aortae from control (Ctrl) and Y/T KO with or without angiotensin II (AngII). (B) Cryosections of mesenteric arteries stained for MAC2 (red), elastin (green), and DAPI (blue). Images represent individual fluorophores (from Figure 7). (C) Cryosections of mesenteric arteries stained for Ki67 (red), elastin (green), and DAPI (blue). Images represent individual fluorophores (from Figure 7).

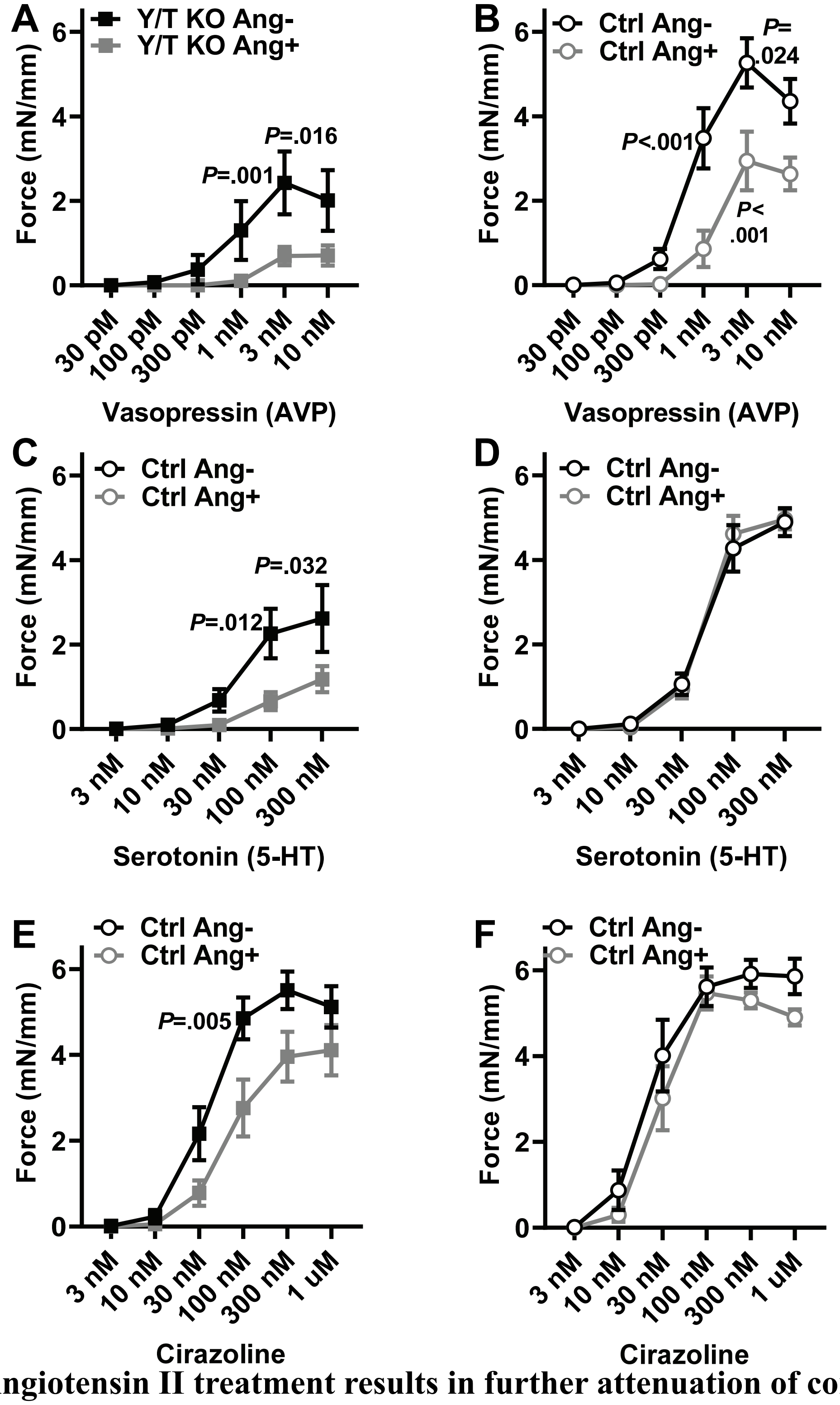

Figure S4. Angiotensin II treatment results in further attenuation of contractile responses in Y/T KO tail arteries. Tail arteries were mounted in a wire myography. The black curves that represent Y/T KO Ang- and Ctrl Ang- are the same data from (Figure 2A-C). Force measurements were integrated over 7 minutes and normalized to the reparations' length. (A) Dose response curve for vasopressin of Y/T KO mice with angiotensin (*n*=8) and without (*n*=8). (B) Dose response curve for vasopressin of control mice with angiotensin (*n*=7) and without (*n*=7). (C) Dose response curve for serotonin of Y/T KO mice with angiotensin (*n*=8) and without (*n*=9). (D) Dose response curve for serotonin of control mice with angiotensin (*n*=8) and without (*n*=9). (E) Dose response curve for cirazoline of Y/T KO mice with angiotensin (*n*=8) and without (*n*=9). (F) Dose response curve for cirazoline of control mice with angiotensin (*n*=9) and without (*n*=9). All data are presented as mean ±SEM.

Major Resources Table

In order to allow validation and replication of experiments, all essential research materials listed in the Methods should be included in the Major Resources Table below. Authors are encouraged to use public repositories for protocols, data, code, and other materials and provide persistent identifiers and/or links to repositories when available.

Animals (in vivo studies)

Species	Vendor or Source	Background Strain	Sex	Persistent ID / URL
Mouse	The Jackson Laboratory	*Wwtr1*^{tm1Hmc} *Yap1*^{tm1Hmc}/WranJ	Male	
Mouse	The Jackson Laboratory	*Myh11*-Cre/ERT2	Male	
Mouse	The Jackson Laboratory	B6.129(Cg)-*Gt*(*ROSA*) *26Sor* $^{tm4(ACTB\text{-}tdTomato,\text{-}EGFP)Luo}$/J	Male	

Antibodies

Target antigen	Vendor or Source	Catalog #	Working concentration	Lot # (preferred but not required)	Persistent ID / URL
Ki67	Abcam	#ab15580	10 ug/ ml (IF)		
YAP/TAZ	Cell signaling	#8418	1:200 (IF), 1:1000 (WB)		
MAC2 (galectin3)	Cedarlane	#CL8942AP	5 ug/ ml (IF)		
SM-MHC	Abcam	#ab53219	1:1000 (WB)		
α-actin	Sigma-Aldrich	#A5228	1 ug/ ml (WB)		
PPARγ	Cell Signaling	#2443	1:500 (WB)		
ROCK1	Cell Signaling	#4035	1:1000 (WB)		
C/EBPα	Cell Signaling	#2295	1:1000 (WB)		
C/EBPβ (LAP)	Cell Signaling	#3087	1:1000 (WB)		
Histone H3	Cell Signaling	#4499	1:1000 (WB)		

Data & Code Availability

Description	Source / Repository	Persistent ID / URL
RNA-sequencing datasets	Gene Expression Omnibus repository, accession code: GSE180631<br>And as supplementary excel file	

Other

Description	Source / Repository	Catalog #
Osmotic mini-pumps	Alzet	Model 2004
Elastic connective tissue stain kit	Abcam	#ab150667
Oil red	Sigma	#O0525
Harris hematoxylin	HistoLab	#01800
Alizarin red	Sigma-Aldrich	A5533-25G
[Arg^{8}] Vasopressin	Tocris	#2935
Serotonin hydrogen maleate	Sigma-Aldrich	#H4511-500MG
Cirazoline hydrochloride	Tocris	#0888

DOI [to be added]

U 46619	Tocris	#1932
Angiotensin II	Tocris	#1158
DMEM Ham's F12	VWR	#L0092-500
2% dialyzed fetal bovine serum	Thermo fisher scientific	#A3382001
Penicillin/streptomycin	Biochrom	#A2212
Insulin	Sigma-Aldrich	#I-5500
Revert 700 Total Protein Stain kit	LI-COR	#926-11010
Nuclear Extraction Kit	Abcam	#ab221978
miRNeasy Mini Kit	Qiagen	# 217084
RNeasy Mini kit	Qiagen	#74104

DOI [to be added]
